# Supplementary material for: Interrogating the Escherichia coli Epitranscriptome Via CRISPR Interference and Nanopore Native RNA Sequencing
Source: Microbiologyopen. 2026 Sep 21;15(5):e70413. doi: 10.1002/mbo3.70413 (PMC13592227; doi:10.1002/mbo3.70413)
Supplement: Supplementary file 1 [file MBO3-15-e70413-s001.pdf]

Interrogating the *Escherichia coli* epitranscriptome via CRISPR interference and Nanopore native RNA sequencing

SUPPLEMENTARY MATERIAL

Miranda E. Pitt[1,2*,§], Jianshu Zhang[2§], An N. T. Nguyen[2], Michael B. Hall[3], Leila Jebeli[4], Leo A. Featherstone[5], Garry S.A. Myers[1,6], Nichollas E. Scott[2] and Lachlan J.M. Coin[2*]

[1]Australian Institute for Microbiology and Infection, University of Technology Sydney, New South Wales, Australia

[2]Peter Doherty Institute, University of Melbourne, Victoria, Australia

[3]UQ Centre for Clinical Research, The University of Queensland, Queensland, Australia

[4]Enteric Diseases Group, Murdoch Children's Research Institute, Victoria, Australia

[5]The Kirby Institute, University of New South Wales, Sydney, Australia

[6]School of Natural Sciences, Macquarie University, Sydney, Australia

*Corresponding authors: Miranda E. Pitt, miranda.pitt@uts.edu.au and Lachlan J.M. Coin, lachlan.coin@unimelb.edu.au

§ These authors contributed equally

TABLES

Table S1. Genes targeted via CRISPRi, PAM site positions, location in genome and score for off-target sites

Gene	Modification, RNA type, position (bp)	gRNA	Sequence (Forward)[#]	Gene length (bp)	Gene position[‡]	PAM start position (bp)[^]	Score (minimal off-targets)[§]
add	-	1[*]	TAGTCAGGAAGCGAGATATTATAC	1002	2383714 - 2384715	+89	0.81
		2	TAGTTCATGGTCGCACTCTTTTTC			+110	0.29
rlmF	m6A, 23S rRNA, 1618	1[*]	TAGTAACGCGACAGGGTAAATATC	927	3215455 - 3216381	-59	0.71
		2	TAGTCATTTCTAAAAACCTTCACA			-92	0.64
rlmJ	m6A, 23S rRNA, 2030	1[*]	TAGTGTGTTCCGGTAAGTAAAAAT	843	237266 - 238108	-29	1.47
		2	TAGTGAGCATGGGTAAAGGTGTTC			-15	0.79
rluD	Ψ, 23S rRNA, 1911/1915/1917	1[*]	TAGTGCGTTGACCGAGTTGGTTTT	981	1280270 - 1281250	+38	0.99
		2	TAGTATATAGTGTGCTATTGTAGC			-70	0.84
rsmF	m5C, 16S rRNA, 1407	1[*]	TAGTCGGGAAATAAACGGTGTGTT	1440	2178852 - 2180291	+4	0.86
		2	TAGTGCAGTTTAGCATAAACGCTC			-54	0.70
rsmG	m7G, 16S rRNA, 527	1[*]	TAGTAGGCAATAAGCTGGTTTTTC	624	4864256 - 4864879	+56	0.92
		2	TAGTAAGCTGGTTTTTCTGGTGAT			+49	0.52

[*]gRNA construct selected for subsequent experiments determined via a combination of best gene position, fewer off-target sites and higher knock down efficiency based on qRT-PCR results.

[#]Reverse gRNA is 5'-AAAC following the reverse compliment of forward sequence. Flanking for insertion via BsaI restriction site.

[‡]Gene positions as per ATCC11775 genome (NZ_CP033092.2) available via NCBI. Represented as start-end position in genome.

[^]PAM (5′-NGG-3′) binding site location represented as before (-) or after (+) start position in gene.

[§]Off target score determined using the crispr-browser.pasteur portal [1]. Higher score indicates fewer off target sites for dCas9 to bind to the genome.

Table S2. Oligos used for qRT-PCR to determine gene knock down efficiency and PCR to confirm gRNA in plasmid

Gene	Sequence (Forward)	Sequence (Reverse)	Alternative oligo
**qRT-PCR**			
add	CTGGGGCGTTAAAGTTCTCG	TACGGGCTGCATCTTCAATG	GACGGGCTGCATCTTCAATG (Reverse)[#,‡]
rlmF	GAGTGGGGCGATTTTTAACG	ATGGCGGGTTACACAAGGTC	
rlmJ	CCGCCGTGGTTTAATCCTTA	CCAGTGGCGAAACGTTTGTA	
rluD	GAAGAAGCGCGTTTTGAACC	ACCAGGTCGCGTGGTTTATT	ACCAGGTCGCGCGGTTTATT (Reverse)[#,‡]
rsmF	GTACCGCCGAGCATTTAAGC	GGTGCATTACCGTCAGCAAA	GGTGCATTATCGTCAGCAAA (Reverse)[‡]
rsmG	TCCTTCGTCAGGTGCAACAT	CGGCTCTGACGGAAACTCTT	CGGCTCTGAAGGAAACTCTT (Reverse)[#,‡]
rpoS[*]	TTGCTGGACCTGATCGAAGA	GAGAAGCGGAAACCACGTTC	TTGCTGGACCTTATCGAAGA (Forward)[#,‡]
rssA[*]	AGGACTAATGGCACCCGTTG	ACGCGTGAGGGAAATAGGAA	AGGACTCATGGCACCTGTTG (Forward)[#,‡]
**PCR**			
pFD152 plasmid[^]	GTCTTTATGAAACACGCATTGA	GGGAATGAGAATAGTGAATGGACC	

[*]Housekeeping genes selected based on prior literature [2,3].

[#]qRT-PCR alternative primer required for isolate ATCC8739.

[‡]qRT-PCR alternative primer required for isolate ATCCBAA2452.

[^]Oligos used to PCR flanking regions of pFD152 with gRNA and sequence checked via Sanger sequencing. To check gRNA was present, pFD152 Forward underwent PCR with gRNA Reverse oligo.

Table S3. Number of significant modification sites detected in ATCC knock down *E. coli* strains against different controls

Strain	Knock down gene	Control*	pFD152[#]
**11775**	*add*	83	731
**11775**	*rlmF*	30	364
**11775**	*rlmJ*	642	3397
**11775**	*rluD*	11	351
**11775**	*rsmF*	1712	708
**11775**	*rsmG*	30	706
**8739**	*add*	1436	4170
**8739**	*rlmF*	3251	10162
**8739**	*rlmJ*	234	7650
**8739**	*rluD*	2025	3181
**8739**	*rsmF*	617	1946
**8739**	*rsmG*	2222	7881

[*]Control contains no pFD152 plasmid (pFD152 (-)).

[#]pFD152 control is pFD152 with activated dCas9 (pFD152 (+), dCas9 (+), no gRNA).

Table S4: Gene positions with unified ggcaller annotation

ggcaller_ID*	Gene	Alt#	11775	8739	25722
sp-E8XH70-LPP1_SALT4	LPP1		477018-477254	2159408-2159644	2381476-2381712
sp-Q8XE41-OMPC_ECO57	OMPC		4761941-4763032	1687186-1688313	1582947-1584077
sp-E0J500-CSPC_ECOLW	CSPC		336248-336508	2008789-2009049	2236106-2236366
sp-A9MT05-EFTU_SALPB (group_388)	EFTU	TUF1, TUF2	2726375-2727559	4456195-4457379	4808415-4809599
sp-A9MT05-EFTU_SALPB (group_381)	EFTU	TUF1, TUF2	3464087-3465316	423510-424739	433585-434814
sp-P0A1R6-DBHA_SALTY	DBHA	HUPA	2703993-2704265	4433689-4433961	4786031-4786303
sp-P0A1R8-DBHB_SALTY	DBHB	HUPB	1688907-1689107	3498197-3498454	3757228-3757485
SP−P0AGA2−SECY_ECOLI	SECY		3491391-3492722	450787-452118	460874-462205

*ggcaller ID includes uniprot protein reference in description.

#Alternative name for gene.

FIGURES

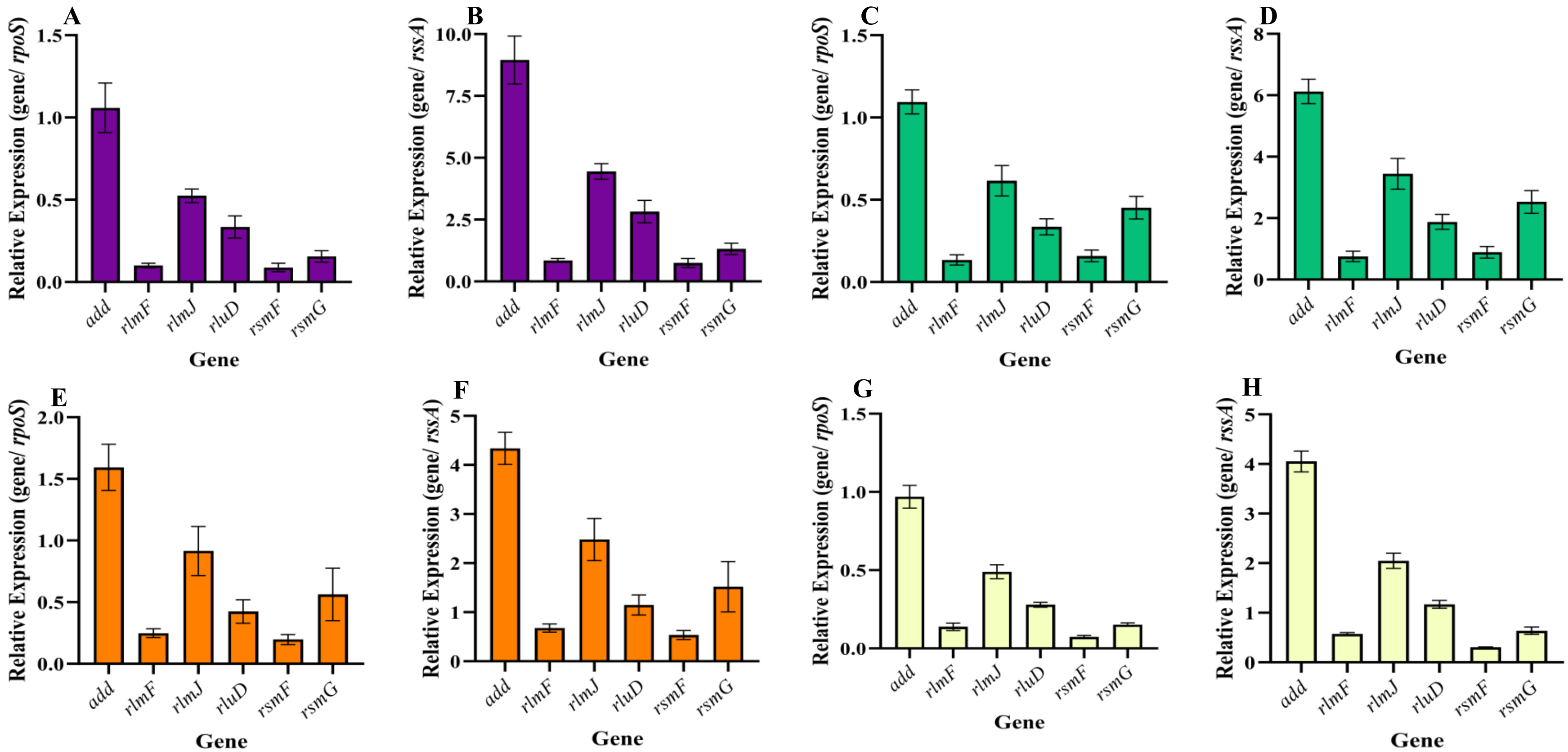

Fig. S1 Expression of six genes across four *E. coli* strains and normalised to two housekeeping genes, *rpoS* and *rssA*. **A** ATCC 11775 *rpoS*. **B** ATCC 11775 *rssA*. **C** ATCC 25922 *rpoS*. **D** ATCC 25922 *rssA*. **E** ATCC 8739 *rpoS*. **F** ATCC 8739 *rssA*. **G** ATCC BAA-2452 *rpoS*. **H** ATCC BAA-2452 *rssA*. Bars represented as average (n=3) ±standard deviation.

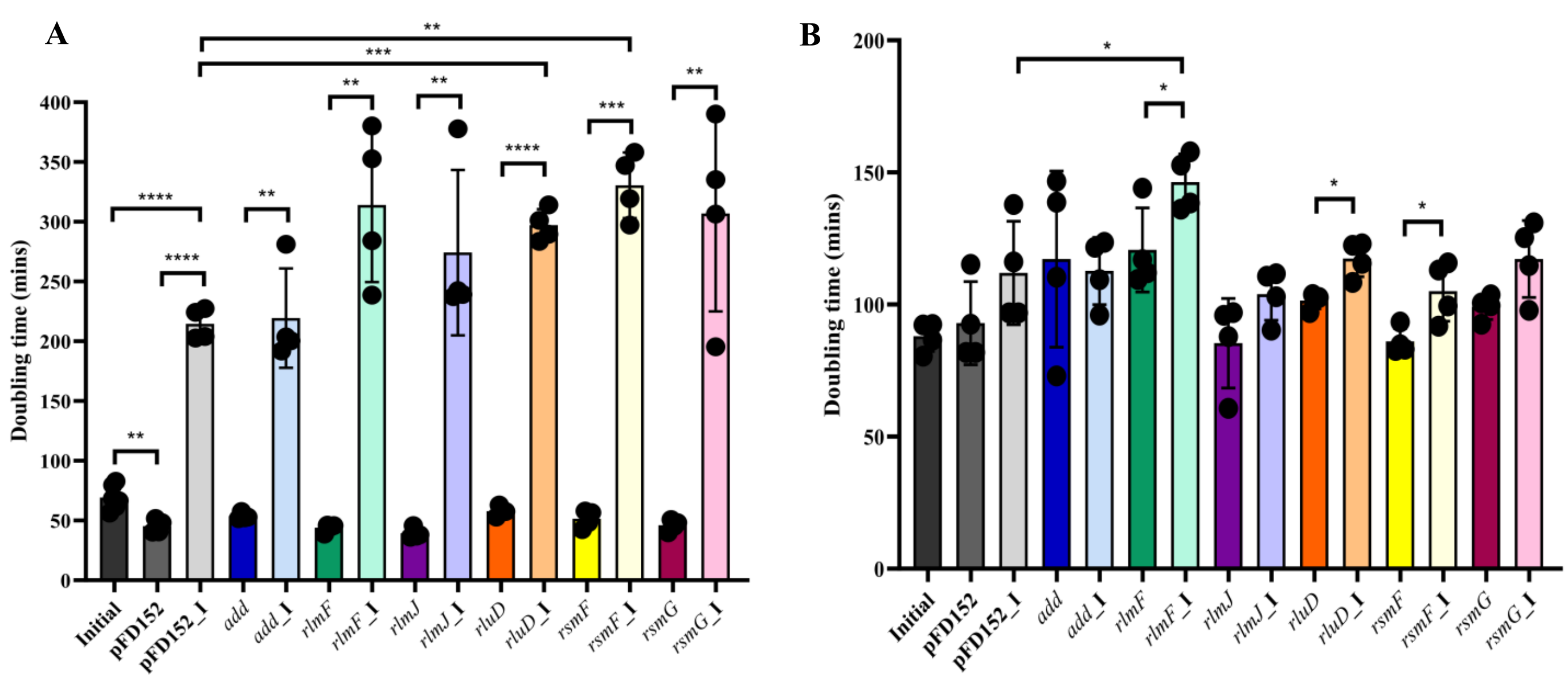

Fig. S2 Growth rate comparison between controls (Initial) and KD (pFD152) for two *E. coli* strains. **A** ATCC 11775. **B** ATCC 8739. Mean±SD, n≥4 biological replicates, "_I" is CRISPRi - activation of dCas9+gRNA. Three controls were included for each strain: "Initial" (first column) represents ATCC strain with no pFD152 plasmid (pFD152 (-)), "pFD152" (second column) is pFD152 within the strain but not activated (pFD152 (+), dCas9 (-), no gRNA) and "pFD152_I" is pFD152 within the strain and activated (pFD152 (+), dCas9 (+), no gRNA). Significance represented as *p<0.05, **p<0.01, ***p<0.001, ****p<0.0001.

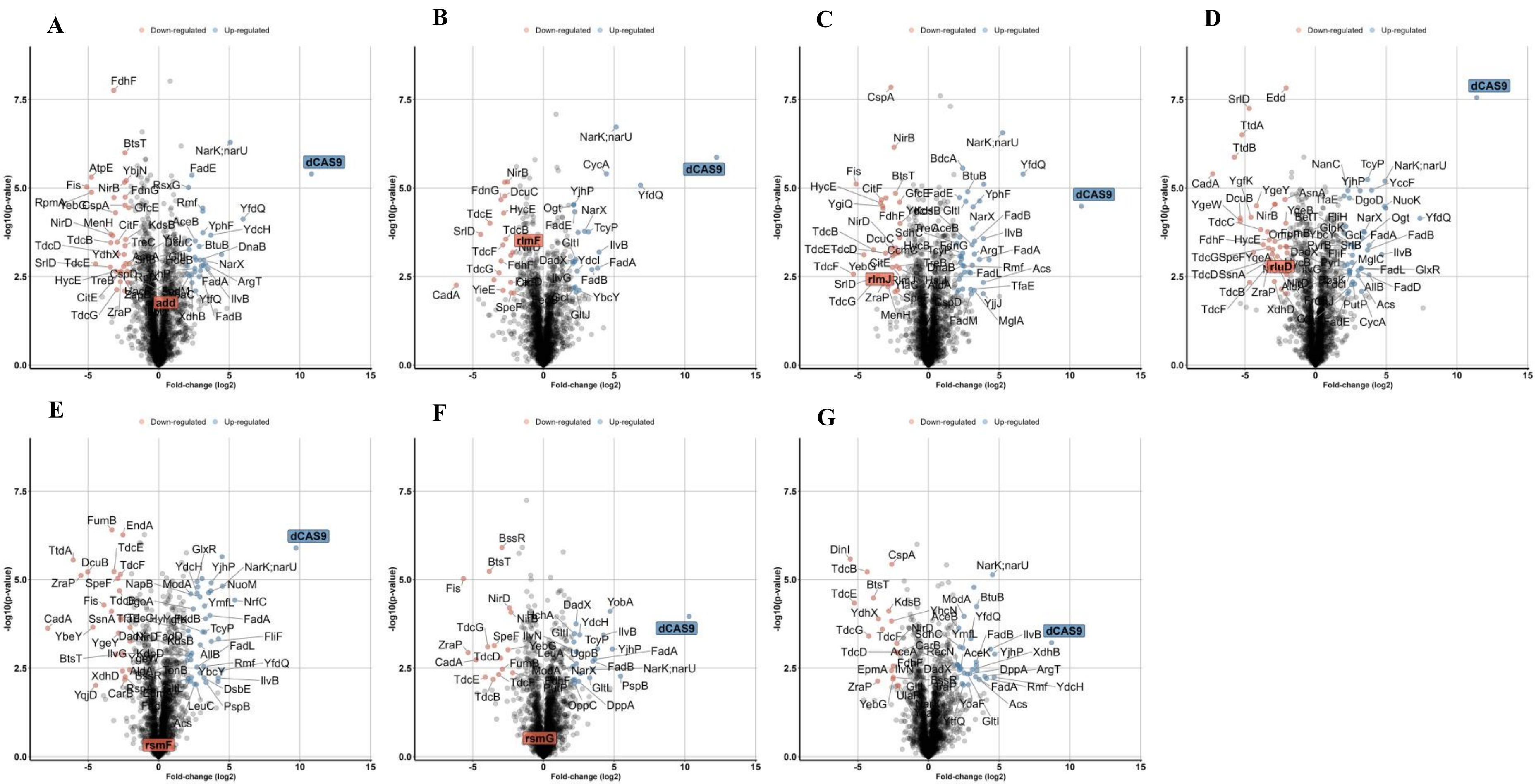

Fig S3 ATCC 11775 protein changes when comparing control (pFD152 plasmid carriage only) and knock down (expression of dCas9 and gRNA) samples. **A** *add*. **B** *rlmF*. **C** *rlmJ*. **D** *rluD*. **E** *rsmF*. **F** *rsmG*. **G** pFD152. Protein levels of genes KD and dCas9 shaded. Down- (orange) and up-regulation (blue) defined as fold-change(log2) >2 or <-2 and -log10 (p-value) >2. n = 4 biological replicates.

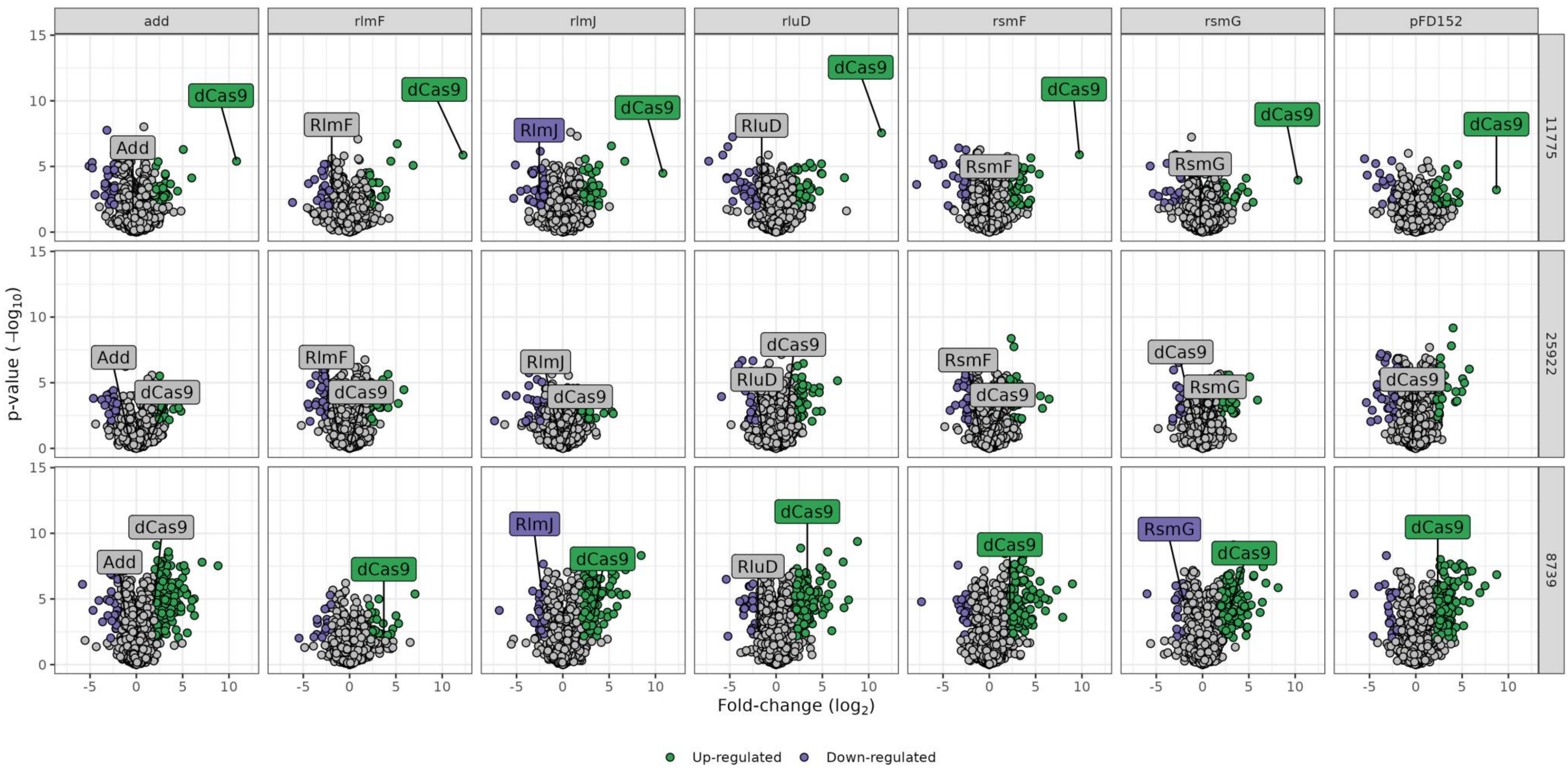

Fig. S4 Protein changes in control with pFD152 plasmid not induced to express dCas9 and gRNA vs pFD152 knock down in three ATCC *E. coli* strains. Down- (purple) and up-regulation (green) defined as fold-change(log2) >2 or <-2 and -log10 (p-value) >2. n=4 biological replicates

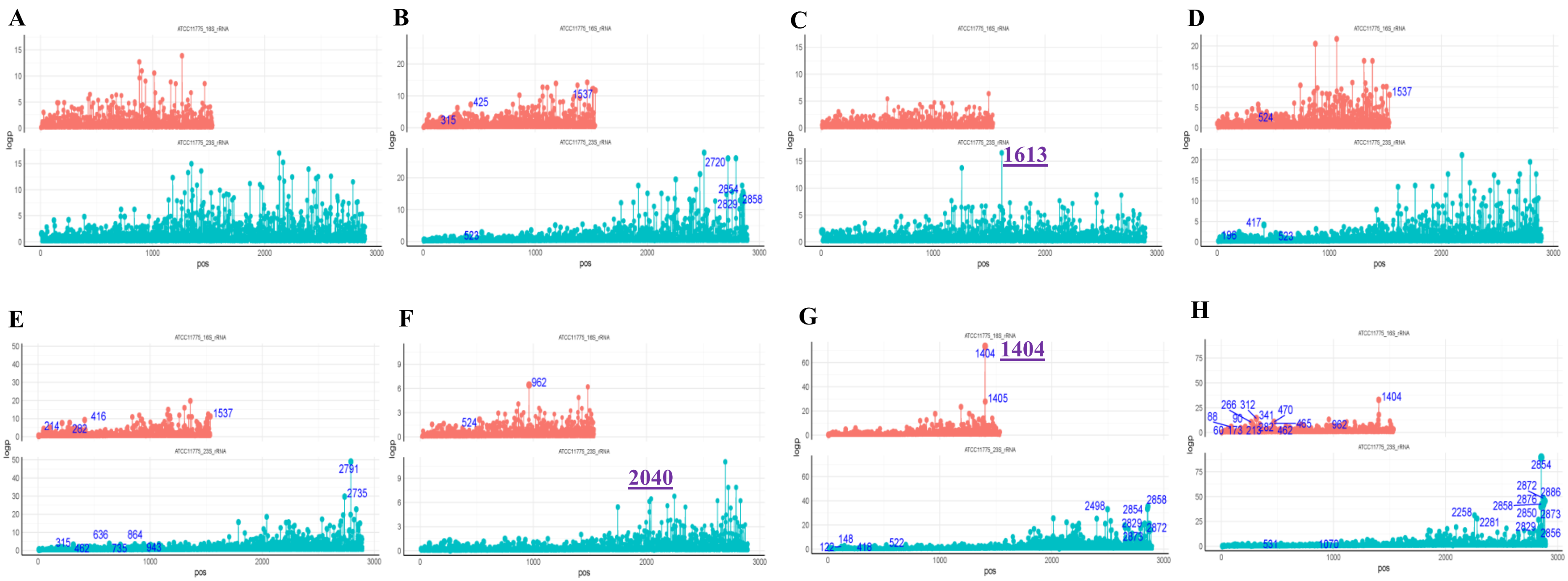

Fig. S5 Nanocompore detection of known rRNA modification sites using flongle (total RNA) data. **A** *add* CRISPRi vs initial control. **B** *add* CRISPRi vs pFD152 control. **C** *rlmF* CRISPRi vs initial control. **D** *rlmF* CRISPRi vs pFD152 control. **E** *rlmJ* CRISPRi vs initial control. **F** *rlmJ* CRISPRi vs pFD152 control. **G** *rsmF* CRISPRi vs initial control. **H** *rsmF* CRISPRi vs pFD152 control. Initial control contains no pFD152 plasmid (pFD152 (-)) and pFD152 control is pFD152 with activated dCas9 (pFD152 (+), dCas9 (+), no gRNA). **Purple bold underlined** position indicates detection of known site.

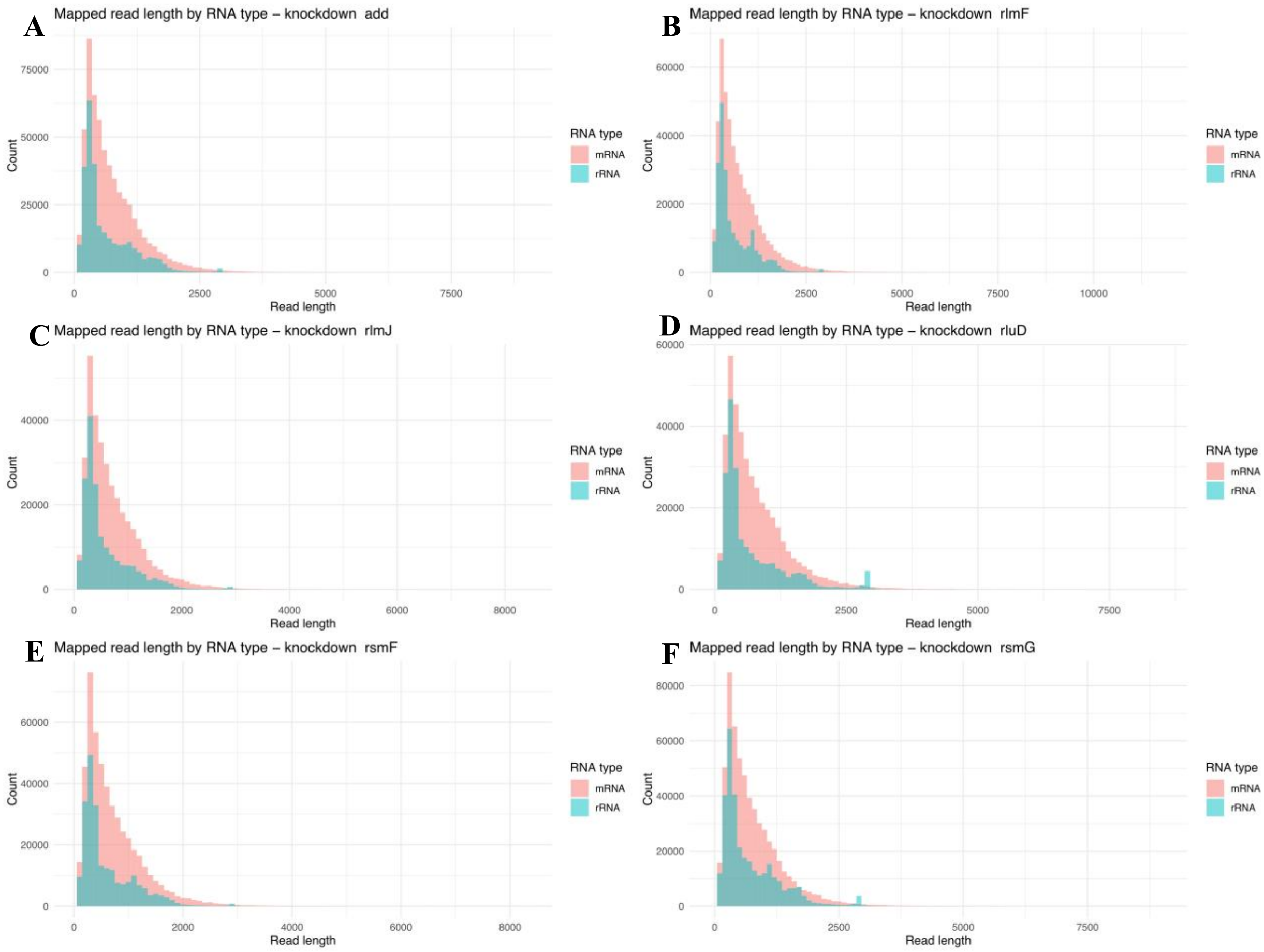

Fig. S6 Nanopore GridION direct RNA sequencing of ATCC 11775 comparing mapped read length for rRNA and mRNA for six CRISPRi samples. CRISPRi samples include: **A** *add*. **B** *rlmF*. **C** *rlmJ*. **D** *rluD*. **E** *rsmF*. **F** *rsmG*.

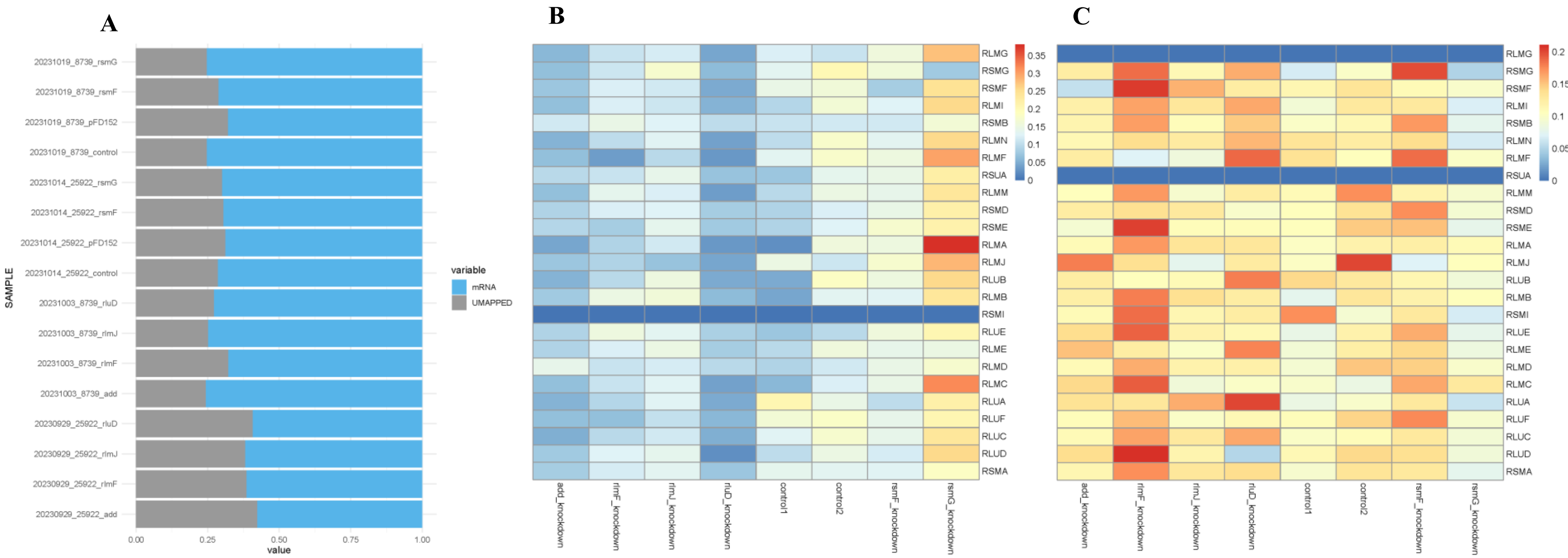

Fig. S7 Nanopore direct RNA sequencing of ATCC 25922 and ATCC 8739 *E. coli* controls and six CRISPRi samples. **A** Proportion of reads mapping to reference (mRNA) for GridION (mRNA enriched) sequencing runs **B** ATCC 25922 heat map identifying repression of genes via CRISPRi detected using Salmon v1.10.1 for mRNA enriched sequencing. **C** ATCC 8739 heat map identifying repression of genes via CRISPRi detected using Salmon v1.10.1 for mRNA enriched sequencing. Control 1 is initial control which contains no pFD152 plasmid (pFD152 (-)) and control 2 is pFD152 with activated dCas9 (pFD152 (+), dCas9 (+), no gRNA).

REFERENCES

1. Cui L, Vigouroux A, Rousset F, Varet H, Khanna V, Bikard D. A CRISPRi screen in *E. coli* reveals sequence-specific toxicity of dCas9. Nat Commun. 2018;9:1912.
2. Jandu N, Ho NK, Donato KA, Karmali MA, Mascarenhas M, Duffy SP, Tailor C, Sherman PM. Enterohemorrhagic *Escherichia coli* O157:H7 gene expression profiling in response to growth in the presence of host epithelia. PLoS One. 2009;4:e4889.
3. Peng S, Stephan R, Hummerjohann J, Tasara T. Evaluation of three reference genes of *Escherichia coli* for mRNA expression level normalization in view of salt and organic acid stress exposure in food. FEMS Microbiol Lett. 2014;355:78-82.
